# Cancer prevention in cancer predisposition syndromes: A protocol for testing the feasibility of building a hereditary cancer research registry and nurse navigator follow up model

**DOI:** 10.1371/journal.pone.0279317

**Published:** 2022-12-22

**Authors:** Holly Etchegary, April Pike, Rebecca Puddester, Kathy Watkins, Mike Warren, Vanessa Francis, Michael Woods, Jane Green, Sevtap Savas, Melanie Seal, Zhiwei Gao, Susan Avery, Fiona Curtis, Jerry McGrath, Donald MacDonald, T. Nadine Burry, Lesa Dawson

**Affiliations:** 1 Clinical Epidemiology, Faculty of Medicine, Memorial University of Newfoundland, St. John’s, Newfoundland, Canada; 2 Faculty of Nursing, Memorial University of Newfoundland, St. John’s, Newfoundland, Canada; 3 Centre for Nursing and Health Studies, Eastern Health, St. John’s, Newfoundland, Canada; 4 Patient Partner, Faculty of Medicine, Memorial University of Newfoundland, St. John’s, Newfoundland, Canada; 5 Division of Biomedical Sciences, Discipline of Oncology, Faculty of Medicine, Memorial University of Newfoundland, St. John’s, Newfoundland, Canada; 6 Discipline of Oncology, Faculty of Medicine, Memorial University of Newfoundland, Cancer Care Program, Eastern Health, St. John’s, Newfoundland, Canada; 7 Family Medicine, Faculty of Medicine, Memorial University of Newfoundland, St. John’s, Newfoundland, Canada; 8 Provincial Medical Genetics Program, Eastern Health, St. John’s, Newfoundland, Canada; 9 Gastroenterology, Faculty of Medicine, Memorial University of Newfoundland, St. John’s, Newfoundland, Canada; 10 Newfoundland and Labrador Centre for Health Information, St. John’s, Newfoundland, Canada; 11 Obstetrics and Gynecology, Faculty of Medicine, Memorial University of Newfoundland, St. John’s, Newfoundland, Canada; GERMANY

## Abstract

Monogenic, high penetrance syndromes, conferring an increased risk of malignancies in multiple organs, are important contributors to the hereditary burden of cancer. Early detection and risk reduction strategies in patients with a cancer predisposition syndrome can save their lives. However, despite evidence supporting the benefits of early detection and risk reduction strategies, most Canadian jurisdictions have not implemented programmatic follow up of these patients. In our study site in the province of Newfoundland and Labrador (NL), Canada, there is no centralized, provincial registry of high-risk individuals. There is no continuity or coordination of care providing cancer genetics expertise and no process to ensure that patients are referred to the appropriate specialists or risk management interventions. This paper describes a study protocol to test the feasibility of obtaining and analyzing patient risk management data, specifically patients affected by hereditary breast ovarian cancer syndrome (HBOC; *BRCA 1* and *BRCA 2* genes) and Lynch syndrome (LS; *MLH1*, *MSH2*, *MSH6*, and *PMS2* genes). Through a retrospective cohort study, we will describe these patients’ adherence to risk management guidelines and test its relationship to health outcomes, including cancer incidence and stage. Through a qualitative interviews, we will determine the priorities and preferences of patients with any inherited cancer mutation for a follow up navigation model of risk management. Study data will inform a subsequent funding application focused on creating and evaluating a research registry and follow up nurse navigation model. It is not currently known what proportion of cancer mutation carriers are receiving care according to guidelines. Data collected in this study will provide clinical uptake and health outcome information so gaps in care can be identified. Data will also provide patient preference information to inform ongoing and planned research with cancer mutation carriers.

## Introduction

### Cancer predisposition syndromes (CPS): Very high cancer risks

Monogenic, high penetrance syndromes, conferring an increased risk of malignancies in multiple organs, are important contributors to the hereditary burden of cancer [1]. Inherited cancers account for up to 10% of all cancers, although variants in cancer predisposition genes can reach a prevalence of 20% in certain cancer types [2, 3]. Over 200,000 Canadians harbor deleterious genetic variants in the >100 genes that cause a CPS, some of which face a 100% risk of developing cancer in their lifetime [4]. Guidelines are available for the management of CPS such as hereditary breast ovarian cancer syndrome (HBOC; *BRCA 1* and *BRCA 2* genes) [5–9] and Lynch syndrome (LS; *MLH1*, *MSH2*, *MSH6*, and *PMS2* genes) [10–13]. High risk individuals are recommended to undergo annual, cancer-type specific surveillance entailing an array of comprehensive clinical exams and imaging such as magnetic resonance imaging (MRI), colonoscopies and mammograms. In CPS where early detection is not possible, prophylactic surgeries are recommended, such as removal of ovaries for carriers of BRCA1/2 genes [5, 8].

#### Early identification and intervention works

Early detection and risk reduction strategies in patients with a CPS can save their lives [4, 14]. Screening effectiveness in reducing morbidity and mortality from colorectal cancer is well supported in LS carriers [15–17]. Follow up of these patients also showed excellent survival following an endometrial and/or ovarian cancer [17]. Preventative gynecologic surgery drastically reduces cancer rates [18, 19]. For female BRCA1/2 carriers, risk-reducing salpingo-oophorectomy (RRSO) decreases the risk of ovarian and breast cancer (BC) by >80% and 50%, respectively, with a 77% reduction in all-cause mortality [20–22]. Annual breast MRI is associated with decreased BC stage, lower overall progression to metastatic disease, and increased survival [23–26], and confers a sensitivity of >90% for early stage BC detection when combined with mammogram [27]. Risk-reducing mastectomy (RRM) nearly eliminates breast cancer risk in female BRCA1/2 carriers and is routinely discussed [28]. In addition to the health and survival benefits for patients, these interventions are cost effective for healthcare systems [29–33]. Some experts suggest that identifying a patient with a CPS *after* a cancer diagnosis is “a failure of cancer prevention” [34].

#### The problem

Despite evidence supporting the benefits of early detection and risk reduction strategies, most Canadian jurisdictions have not implemented programmatic follow up of high-risk patients. This is concerning since the challenges extend beyond patients: their children and other first degree relatives each have a 50% chance of inheriting the same autosomal dominant CPS and timely screening and intervention can save their lives. Unfortunately, follow-up of at-risk family members is often inadequate or nonexistent. This is due to a lack of healthcare provider awareness of CPS [19, 35, 36], workforce shortages in genetic specialists [37], complex and variable referral criteria across provinces [2, 37] as well as provincial differences in screening recommendations. People living in remote areas are isolated from the specialized genetics and oncology clinics typically only available in urban locations. All of this contributes to fragmented and unequitable access to care across Canada, even within CPS families living in different provinces [38, 39].

In our study site in the province of Newfoundland and Labrador (NL), Canada, high-risk individuals receive genetic counseling and testing through the Provincial Medical Genetics Program (PMGP), a single health centre servicing the entire population. If a cancer mutation is identified, patients receive counselling and recommendations in the form of a letter that they are advised to share with their family physician and relatives. Carriers are referred to medical and gynecologic oncology and other specialties as required (e.g., urology). There is no centralized, provincial registry of high-risk individuals. There is no continuity or coordination of care providing cancer genetics expertise and no process to ensure that patients are referred to the appropriate specialists or screening and prevention interventions after they are seen by the PMGP. The quality of care for these mutation-positive patients rests solely on them and their family physicians (for those that have one), and a handful of specialists in the province with an interest in hereditary cancers.

### Possible solutions: Inherited cancer registries, patient navigation, and follow up programs

The World Health Organization recommends that all genetics centers establish registries of genetically determined disease [40]. Canadian recommendations similarly support the implementation of hereditary cancer registries, noting they will 1) improve the identification of high risk individuals; 2) improve their access to appropriate clinical and genetic screening; and 3) improve survival rates for at-risk carrier relatives [41]. Genetic registries of this nature are used in other jurisdictions and have improved patient outcomes [11, 17, 41–47].

A Canadian consensus group reviewed the evidence on registries and summarized their positive clinical outcomes, largely derived from increased enrolment of at-risk individuals who subsequently undergo appropriate cancer screening; high rates of surveillance compliance were observed, with noncompliance rates of less than 5% [41]. Registries have demonstrated a decline in the incidence of cancer [47] and improved survival rates, not only for known carriers, but for their relatives identified as carriers who subsequently undergo targeted screening [17, 41, 47]. While much of the work of Canadian registries has been carried out in research settings, findings have been translated to clinical care to the benefit of patients and families [41]. Long standing research registries in Finland similarly show improved screening and survival in LS families over three generations [48], although high numbers of at risk, untested individuals remain, even in these well-established cohorts. However, registry data reveal that testing and ongoing screening of known mutation carriers is less expensive than no surveillance at all and an efficient use of resources [49, 50]. Registry practices of identifying at risk family members are also cost effective [51, 52], particularly when at-risk family members are managed according to guidelines [52].

### Navigation and follow up for pathogenic variant carriers

The current model of care largely relies on patients and their primary care providers to manage inherited risk, despite providers’ reporting a lack of knowledge, skills and confidence to do this [36, 53]. While all provinces and territories (except Nunavut) have organized, population-based breast screening programs, and most have guidelines for the management of high-risk individuals, only five programs formally manage high-risk women [54]. For example, Ontario has a successful high-risk screening program, staffed with patient navigators whose responsibilities include ongoing patient follow up and management [55]. In NL, the Breast Disease Site Group of Eastern Health (EH) suggests alternating annual MRI and mammography for high-risk women, starting at age 30 [56]. Despite these guidelines, women at high risk of breast cancer in NL are referred back to their primary care provider for management [54]. Furthermore, while most provinces and territories have population-based colon cancer screening programs [57], these generally do not include individuals diagnosed with LS. Rather, these patients are referred to relevant specialists. Again, there is no organized or systematic follow up of these patients and their high-risk relatives.

Patient navigation programs are patient-centered programs that aim to reduce the fragmentation and barriers individuals face navigating the healthcare system [58]. Key principles of patient navigation models, such as ongoing and regular communication and coordination of care throughout the cancer journey [59–61], are relevant to reduce barriers and improve outcomes in individuals with cancer predisposition syndromes (CPS). Nurse navigators play an important role in helping to raise awareness about the importance of hereditary testing and the identification of patients with genetic mutations [59]. A scoping review of navigation models and the roles of navigators in primary care revealed significant variability in roles, scope of practice and models of care, although benefits such as facilitating access to care and navigating the health system and services with patients were common [60]. Oncology patients in navigation programs benefit from strong support, both practical and emotional; having a navigator bridged the gap between diagnosis, screening and treatment, helping to ensure continuity of care [61].

### Study rationale

NL has the highest incidence and mortality rates of colon cancer in Canada and one of the highest rates of familial cancer in the world [62]. Approximately 600 carriers of inherited cancer mutations are known in NL, although clinical experience of the team suggests as many as 1500+ high-risk individuals remain unidentified. It is not known what proportion of NL patients at high risk of inherited cancer access evidence-based screening/prevention interventions or follow recommended guidelines. In known LS carriers, there has been no formal evaluation of the rates of adherence to recommendations for more than a decade [16]. In contrast, BRCA mutation carriers in NL were described up to 2017, with documented rates of adherence to screening interventions and the factors associated with adherence [63]. While risk reducing salpingo-oophorectomy was completed in 76% of eligible women, only 47% had accessed MRI within 12 months as per guidelines. Related qualitative work [64] suggests the sub-optimal MRI uptake was partly due to a lack of coordination of screening. Access to specialist cancer genetics care was the strongest predictor of adherence to inherited cancer risk management guidelines [63]. Our prior work also reveals strong patient support for the creation of an inherited cancer research registry and a desire for a coordinated approach to care and follow up [35, 64, 65]. The protocol described here offers the opportunity to demonstrate the feasibility of obtaining and analyzing patient risk management data, highlight any gaps in adherence to risk management practices and their relationship to health outcomes, and gather crucial patient opinions, before a subsequent application that will focus on creating a registry and follow up navigation model.

### Study objectives

1. Create and populate a database of BRCA and LS mutation carriers in NL
2. Characterize the nature and extent of cancer prevention interventions and cancer outcomes in BRCA and LS carriers and the factors associated with intervention uptake and adherence
3. Determine the priorities and preferences of patients with any inherited cancer mutation for a follow up navigation model of risk management

## Materials and methods

This study was approved by the provincial Health Research Ethics Board and Eastern Health’s Research Proposal Approvals Committee (Ref#: 2022.125).

### Objective 1

Create and populate a patient database of BRCA and LS mutation carriers in NL

#### Design

Retrospective secondary analysis of BRCA and Lynch carriers at the PMGP

#### Population and sampling

Patients identified from 2006 (CPS testing start date) with a BRCA or LS mutation to present day (n = ~600) will be identified by a genetic counselor in the PMGP. The counselor will create a datafile of cancer mutation carriers saved on secure PMGP servers and use secure file transfer to send the file to the provincial data custodian, the Newfoundland and Labrador Centre for Health Information (NLCHI) for linkage.

#### Outcomes

Aggregate-level description of BRCA and Lynch carrier cohorts.

#### Data collection

The following variables will be extracted from the patient database called Shire:

Provincial health card #, pedigree or family # identifier, date of referral, specific cancer mutation identified, date of genetic testing, date of results disclosure, referring physician (whether family practitioner or specialist), laboratory where genetic testing was performed, and whether the patient is the proband (first person to be tested in a family).

#### Analysis

Descriptive statistics including means/standard deviation and counts/percentages will be used to describe continuous and categorical variables, respectively. Descriptions of the overall profiles of BRCA and Lynch patient cohorts will be constructed.

### Objective 2

Characterize the nature and extent of cancer prevention interventions in BRCA and LS carriers and the factors associated with intervention uptake and adherence

#### Design

Population based retrospective cohort of BRCA and Lynch carriers identified through the PMGP in Obj. 1.

#### Population

Patients identified as BRCA and Lynch mutation carriers to present day.

#### Outcomes

(1) Healthcare utilization (uptake of risk management interventions—screening, surgeries, including prophylactic surgery, specialist appointments), (2) adherence to recommended risk management interventions, (3) cancer outcomes (cancer incidence, stage at cancer diagnosis)

#### Data collection

NLCHI will link the data collected from the PMGP in Obj. 1 to other relevant patient data using health card number to form a carrier cohort. The variable list outlining all data to be extracted and linked is in S1 File.

Following linkage and de-identification, NLCHI will provide the file to the study lead (HE) through secure file transfer.

#### Variables to be extracted

We will collect data at least from the year of results disclosure of a CPS mutation (since risk management interventions are subsequently recommended) to present day. When databases allow, we will incorporate data collection 5 years prior to 2006 –the year cancer genetic testing began at the PMGP. This lookback period will allow for statistical control of any prior cancer screening, imaging and surgical risk management practices that are expected to impact risk management after receiving a genetic test result, as well as cancer outcomes.

#### Healthcare Utilization Outcome variables (uptake of risk management interventions)

We will assess the frequency and timing of:

#### BRCA

Prophylactic USO/BSO (unilateral or bilateral salpingo-oophorectomy), prophylactic hysterectomy, prophylactic mastectomy, cancer/malignant related non-prophylactic surgeries, breast MRI, mammograms, transvaginal ultrasound, breast ultrasound (potentially used for diagnostic purposes), PSA screening for male carriers, # and type of specialist appointments.

#### Lynch

Prophylactic USO/BSO (unilateral or bilateral salpingo-oophorectomy), prophylactic hysterectomy, prophylactic colectomy, cancer/malignant related, non-prophylactic surgeries, endometrial biopsy, colonoscopy, endoscopy, FIT testing, # and type of specialist appointments.

For both carrier groups, we will also collect data on prior cancers, comorbidities such as diabetes or heart disease, medication usage, and demographic information such as sex, rural/urban residence, age, parity.

#### Adherence to risk management interventions

Eligibility criteria for each risk-reducing intervention for all carriers will be strictly defined according to guidelines [9–13]. We will then categorize each patient’s adherence to risk management interventions as not adherent, somewhat adherent, or very adherent [63]. There is no gold standard definition for compliance with high risk screening and surgical interventions in CPS patients. Clinical expertise on the team and input from patient partners suggested that some patients undergo some, but not all interventions, while some undergo all and a lesser number undergo none. We want to describe this variability in uptake, and identify whether some interventions have higher uptake than others and could benefit from targeted intervention by the nurse navigator in our eventual feasibility study. Adherence will be measured for each patient at one point in time, the latest that available data allows. We will have a lookback period of up to five years where databases allow (but at least one year) to account for colonoscopy screening recommendations of 1–5 years and to allow flexibility around scheduling breast MRI within menstrual cycles as in our prior work [63].

#### Cancer outcomes

We will use the provincial cancer care registry to assess incident cancers diagnosed over time after genetic testing, and cancer stage at diagnosis for all BRCA and Lynch carriers.

#### Predictor variables

Sex, age, rural/urban, prior history of cancer, mutation (BRCA or LS), time since receiving genetic testing results, and having specialist assessment. We will calculate driving time to a specialist genetics clinic (St. John’s), as we know patients in rural and remote NL face challenges in accessing genetics care and expertise [35, 64] and this expertise is related to risk management uptake [17, 47, 63].

#### Covariates

Referring physician (family physician or specialist), parity, comorbidities, prior risk management interventions such as cancer screening, surgeries, smoking history.

#### Data analysis

1) Frequencies/proportions and means/standard deviations will be used to describe categorical and continuous variables, respectively. Multinomial logistic regression will be used to identify significant predictors of our primary outcome variables: adherence to recommended risk management interventions (not adherent, somewhat adherent, or very adherent) and the stage of cancer diagnosis (no cancer diagnosis, early (stage I-II), and late (stage III-IV) in univariate and multivariate analysis. We will use logistic regression to identify predictors of cancer incidence. Poisson regression will be used to identify significant predictors of the number of cancer screens over time. In the case of over-dispersion in Poisson regression, Negative Binomial regression will be used. Generalized linear models with Generalized Estimating Equations (GEE) will be used to adjust for possible correlation among individuals from the same family. The purposeful selection method will be used to build our final models. Statistical analyses will use SPSS version 28 (IBM Corp.) with p < 0.05 considered statistically significant.

#### Hypotheses

We expect lower uptake of risk management interventions in carriers from rural NL (due to driving time to access screening and testing) and younger ages (e.g., lower prophylactic surgical uptake is expected in younger women who perhaps have not completed childbearing). We expect higher uptake of risk management interventions in carriers with a personal history of cancer, in keeping with our prior analyses [63] and other work [66, 67]. We expect carriers with better adherence to have lower incidence of cancer and earlier stage cancer. We expect carriers cared for by specialists with cancer genetics expertise to have better adherence to risk management strategies than those carriers not seen by specialists with cancer genetics expertise, even when controlling for other predictors and covariates. In NL, these specialists are few and known to the team. Cancer genetics specialist counselling (outside of genetics clinics) has been associated with higher risk management uptake in high-risk patients in other jurisdictions [68, 69].

### Objective 3

A patient-oriented, qualitative interpretative descriptive (ID) [70] study will explore the priorities and preferences of cancer mutation carriers. This data will guide the development of a nurse-led patient navigation model of risk management–the focus of an upcoming grant application. Previous research has identified that these patients in NL face systemic barriers in their adherence to inherited risk management [19, 35, 64]. However, no research has been conducted to date where these carriers identified their priority needs and preferences for a risk management program, and very little research has included carriers of cancer mutations beyond *BRCA* and Lynch syndrome (e.g., gastric cancer mutations or moderate penetrance genes such as *RAD51C*, *ATM)*. It would be futile to implement a navigation and follow up model of care with features imposed by researchers, without the input of these patients.

### Qualitative design

Using a qualitative interpretative descriptive (ID) approach [70], semi-structured interviews will be conducted with cancer mutation carriers. ID is an approach developed for use in the applied disciplines. Its origins are within the discipline of nursing [71] and is appropriate when the goal is to develop evidence to inform clinical practice and policy. This approach is well-suited to our objective, which is to understand the experiences and preferences of CPS carriers for the purpose of informing how a nurse-led navigation program is best poised to meet their unique needs. The ID author developed several evaluative criteria for ID studies [70] (e.g., representative credibility, analytic logic, interpretive authority) that we will use to guide our study approach.

Inclusion criteria:

- Patients living in NL with or without a history of cancer with any molecularly confirmed hereditary cancer syndrome [HCS] (any one or a combination of any hereditary cancer genes including *LS* genes, *BRCA1/2*, *ATM*, *CHEK2*, *PALB2*, *RAD51C*, *CDH1*, *MEN1*, *VHL*, etc.)
- 18 years old or older

Exclusion:

- Unable to provide informed consent or life situation suggesting an interview would be emotionally upsetting or burdensome, as determined by providers within patients’ circle of care

#### Sampling

The ID quality criterion of representative credibility [70] will be achieved through appropriate sampling [72, 73]. Our use of purposive and maximum variation sampling will ensure that data capture a broad, equitable range of lived experiences with CPS (e.g., age, sex, gender, region, education, length of time since receiving genetic test result). Our prior, related work [35] suggests up to 40 interviews should capture meaningful patterns in participants’ varied experiences. This estimate gives us confidence that this sampling target will adequately address our research question and our aim to generate usable findings for policy and clinical practice. Providers within these patients’ circle of care will invite them to participate in the study, whether during regular appointments, by phone or email. These include genetic counselors in the PMGP, as well as several clinical team members who provide care to mutation carriers in the province.

The qualitative portion of the study will also be advertised widely over social media and communication channels of Memorial University and the regional health authorities in NL, as well as traditional media such as local radio; we will post the study poster in hard copy in community settings (e.g., St. John’s Farmer’s Market, grocery stores, etc.) wherever permissible.

The names of interested patients will be given to specified team members (RP, AP) via secure email or by telephone who will make contact, explain the purpose of the study, and arrange an interview.

### Data collection and management

Interviews of up to one hour on average will be done by two team members (RP, AP) experienced in ID via telephone, online platform, or in person at a time convenient for participants. Participants will receive a small financial incentive to remove any barriers from participating and to show appreciation for their time and input. Consistent with a patient-oriented approach, the final interview guide has been co-created with patient partners (S2 File). To ensure the ID quality criterion of interpretive authority [70] is met, interviews will be audio-recorded, transcribed verbatim, and imported into NVIVO, a qualitative data analysis software. The use of these techniques will enhance the trustworthiness of our data interpretations. To further assure credibility of the researchers’ interpretive authority, the two researchers will keep reflexive journals [70, 71] throughout the data collection and analysis processes.

#### Informed consent procedures

Once participants contact the team or the team receives a name from a study recruiter, the initial verbal conversation with potential participants will explain the study and offer the first chance to answer any questions. A study consent form will be forwarded to them either via e mail or regular mail as per their preference and an interview time scheduled if they express an interest in participating during the first conversation. During the initial conversation, they will also be told the interviews will be audio taped for later transcription.

Both verbal and written consent will be obtained as approved by the provincial research ethics board. At the start of the interview, the interviewer will review consent forms with participants, answer any questions and remind them that taking part in no way will affect their healthcare. If after reviewing the consent the participant would still like to take part in the study, interviewers will ask them to demonstrate consent with a verbal yes to the question “Is this all agreeable to you?” and make sure there are no further questions. A discussion about preference for returning participant consent forms will be had at that time as well (emailed back to the team or a stamped return envelope mailed to the participant for hard copy return). Interviewers will sign the consent form which will be kept in a separate location from qualitative transcripts and data in locked study offices at Memorial University.

If the participant consents for the interview to be audio-recorded, this will include informing participants that audio recordings will be transcribed by an external transcription service or transcriptionist. De-identified transcripts will be securely transferred to the relevant study team members and stored on a Memorial University computer with university domain level password protection.

At the end of the interview, participants will be given the option to leave an email address with interviewers so they can receive a copy the study findings and/or information about end of study presentations of findings.

### Data analysis

The examination of the transcripts will follow a thematic analysis approach [74] in which textual data is coded and labeled in an inductive manner. This process of coding is iterative, with data analysis using the constant comparison method occurring alongside the interviews [74, 75]. As such, data analysis will be ongoing in parallel to the interviews, allowing us to modify future interviews should new themes be developed that were not part of the original schedule. This approach will allow for the revision, combination or separation of codes in light of new data [75, 76]. As we are taking a comparative approach, comparing patient preferences and experiences, rather than solely describing individual experiences, each newly coded incident will be compared both within and across cases to previous incidents in order to refine or revise the code [77]. After an initial phase of open coding, individual codes will be grouped into overarching themes or constructs through a process of data reduction. Consequently, the theme operates at a higher level than the immediate codes. Interviews will be coded independently by two researchers who will discuss between themselves, before presenting analyses to patient partners and the study lead for reflexive team debriefing [78]. As decisions about data reduction are being made, a transparent, defensible audit trail will be kept of these decisions during the data analysis process to ensure that analytic logic [70] is maintained during data analysis.

### Data reporting

In line with ID [70], in our final themes, we will report the who, what, and where of carriers’ preferences for features of a nurse-led navigation program (e.g., acceptability of a nurse navigator, nature/frequency of screening reminders, other information/services desired, etc.). Beyond the who, what, and where, we will also uncover the ‘so what?’ [70]. In other words, ‘what do these findings mean to policy and practices for the healthcare of this high-risk population?’ Representative credibility [70] will also be maintained in our data reporting, through thick description of participant quotes [79, 80] that support the interpretive claims being made.

### Data collection timelines

Qualitative data collection will begin in Feb-March 2023. At the time of writing, quantitative data collection had begun (September 2022); it ss expected to take up to 6 months to receive the final, linked datafile.

## Discussion

### Privacy and confidentiality considerations

All information collected during this study, including personal information, will be kept confidential and will not be shared with anyone outside the study unless required by law. Study data will have identifiable information removed before analysis (e.g., the qualitative transcripts, the de-identified datafile received from NLCHI). Each participant who completes an interview and their responses (data) will be assigned a specific code and only relevant study team members will have access to the code key which can link the codes back to the qualitative data. The code key will be kept in a password-protected file on a MUN computer with university domain level password protection in locked offices.

The interviews will be recorded and transcribed with the participant’s agreement through the consent process. Participants will be encouraged to not use the names of persons or identifiable place or dates in the qualitative interview to further protect their privacy and confidentiality.

All audio recordings will be destroyed after they have been transcribed and verified for accuracy by the study team. Any identifying information such as names of individuals, institutions, or geographic locations will be removed from the transcripts by the study team during the process of transcript verification. Recording devices (digital recorders for telephone or in person interviews) are password protected and will kept in a locked drawer in the study offices of the interviewers. Recording devices are only accessible by relevant study team members. Immediately after an interview has been completed, all recordings will be saved on a Memorial University computer with university domain level password protection in locked offices. Subsequently the interview will be erased from the recorder or the online platform (Webex) account.

The quantitative data will be de-identified in an SPSS file before it is returned via secure file transfer. NLCHI staff will remove health card #s and Pedigree #s from the final, linked file and replace these with a study ID # for each patient. The datafile will be stored on a Memorial University computer with university domain level password protection accessible only by team members analyzing the dataset.

### Knowledge translation and dissemination plan

We are guided by an integrated knowledge translation approach, engaging with stakeholders from the time of project initiation and throughout via the large multidisciplinary team including patient partners. We have 5 target audiences for dissemination: researchers, patients, the general public, healthcare providers, and decision makers. General public and patients: we will host virtual town halls to present study results and make summaries of study findings to post on social and traditional media. Researcher, provider and decision maker audiences: We will use traditional methods including publication in relevant academic journals and presentations at conferences and grand/specialty rounds, as well as health authority presentations and meetings with relevant decision makers. Finally, findings from the qualitative study will be used to inform the development of tailored, patient-centered education for healthcare providers who provide care to this population.

### Challenges and mitigation strategies

#### Chart & database reviews

Data at the PMGP exist in paper charts and Shire, a database few outside of PMGP have used. We have two experienced genetic counselor collaborators and a research assistant on this project who will be crucial to extract data appropriately. Administrative data: Administrative data have the potential for errors and missing data. Missing data can be addressed using statistical techniques (e.g. imputation). Multivariable regression modeling can adjust for covariates that may influence the uptake of risk management practices (e.g., prior cancer) to minimize confounding. Some limitations are outweighed by the strengths of administrative data: the ability to access data on the provincial cohort of carriers and less susceptibility to recall bias than patient self-reported measures. We have also had many planning discussions with NLCHI and the PMGP in an effort to capture the necessary data to answer study questions, as well as ensure data is available. Qualitative methods: Qualitative methods do not produce generalizable findings. However, they provide experiential, context-specific data on patients’ needs and experiences that can’t be accessed through other methods, which can align policy and clinical practice.

#### Sex and gender considerations

Sex as a biological variable and gender identity, as well as socially-gendered roles in genetics are considered throughout our design and team. Team: Our team is comprised of a mix of males and females of varying career stages from student to honorary professor. Recruitment: Purposive, maximum variation sampling of participants for the qualitative study (Obj3) helps ensure data reflect a broad range of perspectives (e.g., age, sex, gender, ethnicity, region, education). Qualitative data: Interviews will consider the gender identity of the interviewer and the interviewee, which can influence the dynamics of the interview [81]. Socially gendered roles in genetics can impact how participants engage with genetic testing/risk management decisions or results communication (e.g., women are often the gatekeepers of genetic information in the family, more likely to engage with testing and screening) [82–84]. In our data, we will consider the influence of gender identity and socially gendered roles (e.g., standard care for individuals with cancer predisposition affecting biological sex organs can be standardized to cis-gendered persons and this may have implications for the engagement of LGBTQ2A+ persons in screening and follow-up) [85].

#### Quantitative data

Analysis will account for biological sex and self-identified gender across all outcomes. Negative results with respect to sex and gender will be reported. KT: We will also take any sex or gender differences into account in the creation of study outputs as it is possible there will be differences to highlight (e.g., different recommendations for increasing screening uptake among sexes or policy recommendations accounting for gendered preferences in a nurse navigation model of care).

#### Significance of proposed work

The prevalence of pathogenic variants in known cancer predisposition genes is almost 2% in the general population [86]. Cancer control and improved clinical management of high-risk individuals is urgently needed if cancer is to be prevented. Prospective studies of mutation carriers show excellent survival with appropriate risk management [17]. In NL, it is not yet known what proportion of cancer mutation carriers are receiving appropriate screening and other risk management. The data collected in this study will provide crucial clinical uptake and health outcome information so gaps in care can be identified. Data will also provide patient preference information to inform our ongoing research with cancer mutation carriers.

## Supporting information

S1 File Variables to be extracted for cohort study. (DOCX)

S2 File Qualitative interview guide. (DOCX)
