# Supplementary material for: Cancer prevention in cancer predisposition syndromes: A protocol for testing the feasibility of building a hereditary cancer research registry and nurse navigator follow up model
Source: PLoS One. 2022 Dec 22;17(12):e0279317. doi: 10.1371/journal.pone.0279317 (PMC9778977; doi:10.1371/journal.pone.0279317)
Supplement: S1 File — (DOCX) [file pone.0279317.s001.docx]

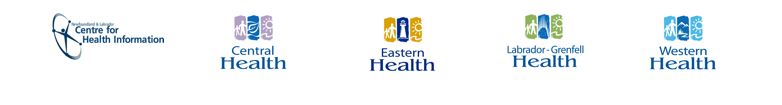


**TITLE of PROJECT:** Cancer prevention in cancer predisposition syndromes: Testing the feasibility of building a hereditary cancer research registry and nurse navigator follow up model **NAME OF PRINICPAL INVESTIGATOR (PI):** Holly Etchegary **ORGANIZATION OF PI:** Memorial University **ROLE:** Associate Professor Faculty of Medicine **EMAIL ADDRESS OF PI:** holly.etchegary@med.mun.ca **TELEPHONE NUMBER OF PI:** 709-864-6605 (office); 709-770-0253 (cell) **NAME AND EMAIL ADDRESS OF KEY CONTACT (IF DIFFERENT FROM THE PI):**

Data Custodian Reason for Data Collection

■ Newfoundland and Labrador Centre for Health Information ■Secondary Use/Chart Review

■Eastern Health ☐General Research

☐Central Health ■Genetic/Genomic Research

☐Western Health ☐Clinical Trial

☐Labrador-Grenfell Health ☐Program Evaluation

☐Other ☐Other ­________________________

**Provide a complete and accurate list of all data elements/fields that you require along with rationale that explains why you require each requested element/field. When specifying the data elements/fields, please indicate which date/year ranges are required. If you have any questions while completing this form please contact** [**DataAccess@nlchi.nl.ca**](mailto:DataAccess@nlchi.nl.ca)**. Note this Variable List must be submitted with the HREB application.**

| **Data Custodian** | **Name of Requested Database** | **Variable(s) Requested** | **Rationale** | **Date/Year Range of Data Requested** |
| --- | --- | --- | --- | --- |
| NLCHI |  | Unique Study ID | To provide de-identified data to the requestor.  Linkages between EH and NLCHI data will be performed by NLCHI using MCP number and will be replaced by a unique ID before being sent to the requestor. | 2001 - most recent data available |
| NLCHI | Client Registry | Year of birth | Year of birth will be used along with date of genetic testing and date of diagnosis to describe the age of patients at the time of testing and diagnosis | 2001-most recent data available |
| NLCHI | Client Registry | Sex | To describe the population of patients who have undergone genetic testing for hereditary cancer | 2001-most recent data available |
| NLCHI | Master Geography File | Census division | To allow description of the place of residence of patients who have undergone genetic testing for hereditary cancer | 2001-most recent data available |
| NLCHI | Master Geography File | Regional Health Authority of Residence | To allow description of the place of residence of patients who have undergone genetic testing for hereditary cancer | 2001-most recent data available |
| NLCHI | Mortality System and Client Registry | Flag to indicate deceased | To allow description of the impact of risk management interventions on mortality | 2001-most recent data available |
| NLCHI | Mortality System and Client Registry | Year of death | To allow exploration of impact of risk management on mortality | 2001-most recent data available |
| Eastern Health, Provincial Medical Genetics Program | Shire | MCP# of patients who have undergone genetic testing | To allow linkage of patients’ data in Shire to other NLCHI and EH databases (MCP# is not needed by the study team, it is only used to allow data linkage from information in Shire) | 2006-most recent data available |
| Eastern Health, Provincial Medical Genetics Program | Shire | Pedigree # | To allow data checking/patient identity confirmation if ever necessary by counselors in the PMGP | 2006-most recent data available |
| Eastern Health, Provincial Medical Genetics Program | Shire | Date of referral to Provincial Medical Genetics Program | To describe wait time between referral to the PMGP and results disclosure and explore whether there is a relationship between this time and post-test outcomes | 2006-most recent data available |
| Eastern Health, Provincial Medical Genetics Program | Shire | Date of genetic testing | To describe wait time between test and results disclosure and explore whether there is a relationship between this time and post-test outcomes | 2006-most recent data available |
| Eastern Health, Provincial Medical Genetics Program | Shire | Specific hereditary cancer mutation(s) identified | To describe testing results and analyse the relationship between results and post-test clinical and health utilization outcomes | 2006-most recent data available |
| Eastern Health, Provincial Medical Genetics Program | Shire | Date of results disclosure | To describe wait time between test and results disclosure and explore whether there is a relationship between this time and post-test outcomes | 2006-most recent data available |
| Eastern Health, Provincial Medical Genetics Program | Shire | Referring physician – GP or Specialist  If specialist, name of specialty (e.g., oncology, neurology) | To analyse predictors of genetic testing and explore impact of referring physician on outcomes | 2006-most recent data available |
| Eastern Health, Provincial Medical Genetics Program | Shire | Laboratory where testing was performed | To describe the process of hereditary cancer genetic testing | 2006-most recent data available |
| Eastern Health, Provincial Medical Genetics Program | Shire | Category of patient having genetic testing: Proband or relative | To describe genetic testing population and explore predictors of outcomes | 2006-most recent data available |
| NLCHI | NLPDP  &  Pharmacy network | Generic Name and DIN | To allow analysis of the impact of genetic testing on any medication/prescription changes. | 2007 - most recent data available |
| NLCHI | NLPDP  &  Pharmacy network | Date of first dispense | To allow analysis of the impact of genetic testing on any medication/prescription changes. Date of the first dispense will establish a timeline of medication use in relation to genetic testing. | 2001 - most recent data available |
| NLCHI | NLPDP  &  Pharmacy network | Date of last dispense | To allow analysis of the impact of genetic testing on any medication/prescription changes.  Date of the last dispense will establish a timeline of medication use in relation to genetic testing. | 2001 - most recent data available |
| NLCHI | Live birth system  &  Stillbirth system | Parity | The reproductive history will be controlled for in analyses of the impact of genetic testing on subsequent clinical outcomes such as reproductive system cancers. | 2001-most recent data available |
| NLCHI | Live birth system  &  Stillbirth system | Gravidity | The reproductive history will be controlled for in analyses of the impact of genetic testing on subsequent clinical outcomes such as reproductive system cancers. | 2001-most recent data available |
| NLCHI | Live birth system  &  Stillbirth system | Smoking history | Smoking history will be controlled for in analyses of the impact of genetic testing on subsequent clinical outcomes such as clinical diagnoses (cancers, heart disease, etc.) | 2001-most recent data available |
| NLCHI | Chronic Disease Registry/CCDSS | Diabetes | Prior clinical diagnoses will be used as predictors or potential confounds in regression analyses of genetic testing  Any clinical diagnoses post genetic testing results disclosure will also be analyzed as potential impacts of genetic testing | 2001 – most recent data available |
| NLCHI | Chronic Disease Registry/CCDSS | Hypertension | Prior clinical diagnoses will be used as predictors or potential confounds in regression analyses of genetic testing  Any clinical diagnoses post genetic testing results disclosure will also be analyzed as potential impacts of genetic testing | 2001 – most recent data available |
| NLCHI | Chronic Disease Registry/CCDSS | AMI | Prior clinical diagnoses will be used as predictors or potential confounds in regression analyses of genetic testing  Any clinical diagnoses post genetic testing results disclosure will also be analyzed as potential impacts of genetic testing | 2001 – most recent data available |
| NLCHI | Chronic Disease Registry/CCDSS | Ischemic Heart Disease | Prior clinical diagnoses will be used as predictors or potential confounds in regression analyses of genetic testing  Any clinical diagnoses post genetic testing results disclosure will also be analyzed as potential impacts of genetic testing | 2001 – most recent data available |
| NLCHI | MCP Fee-For Service & PDAD & Meditech Laboratory Data | Diabetes diagnosis | Prior clinical diagnoses will be used as predictors or potential confounds in regression analyses of genetic testing  Any clinical diagnoses post results disclosure will also be analyzed as potential impacts of genetic testing | For years where CDR/CCDSS is not available |
| NLCHI | MCP Fee-For Service & PDAD | Hypertension | Prior clinical diagnoses will be used as predictors or potential confounds in regression analyses of genetic testing  Any clinical diagnoses post results disclosure will also be analyzed as potential impacts of genetic testing | For years where CDR/CCDSS is not available |
| NLCHI | MCP Fee-For Service & PDAD | Ischemic Heart Disease | Prior clinical diagnoses will be used as predictors or potential confounds in regression analyses of genetic testing  Any clinical diagnoses post results disclosure will also be analyzed as potential impacts of genetic testing | For years where CDR/CCDSS is not available |
| NLCHI | PDAD | AMI | Prior clinical diagnoses will be used as predictors or potential confounds in regression analyses of genetic testing  Any clinical diagnoses post results disclosure will also be analyzed as potential impacts of genetic testing | For years where CDR/CCDSS is not available |
| NLCHI | PDAD | Prophylactic surgeries:  Prophylactic USO/BSO (unilateral or bilateral salpingo-oophorectomy)  Prophylactic hysterectomy  Prophylactic mastectomy  Prophylactic gastrectomy  Prophylactic colectomy | Any surgeries post results disclosure will be analyzed as potential impacts of genetic testing. | 2001-most recent data available |
| NLCHI | PDAD | Year and month of any prophylactic surgeries | To describe potential impact of genetic testing | 2001-most recent data available |
| NLCHI | PDAD | Any oncology surgeries involving malignancies (not classified as prophylactic):  For example, mastectomy, breast reconstruction, removal of dermatological malignancies, colectomy, etc.) | Any surgeries post results disclosure will be analysed as potential impacts of genetic testing | 2001-most recent data available |
| NLCHI | PDAD | Year and month of any oncology/malignant surgery not classified as prophylactic | To allow description of the impact of genetic testing | 2001-most recent data available |
| NLCHI | PDAD | Core biopsies | To allow description of the impact of genetic testing | 2001-most recent data available |
| NLCHI | PDAD | Year and month of any core biopsies | To allow description of the impact of genetic testing | 2001-most recent data available |
| NLCHI | PDAD | Hereditary cancer screening behaviors:  Colonoscopy  Sigmoidoscopy  Endoscopy  Endometrial biopsy | Number pre and post genetic testing  To describe surveillance behaviors pre- and post- sequencing and explore genetic testing impact on post surveillance behavior | 2001-most recent data available |
| NLCHI | PDAD | Year and month of all screening behaviors outlined above | To describe surveillance behaviors pre- and post- sequencing and explore genetic testing impact on post surveillance behavior | 2001-most recent data available |
| NLCHI | Meditech - Medical Imaging | Mammogram  Breast MRI  Transvaginal ultrasound  Breast ultrasound | Number pre and number post genetic testing  To describe imaging behaviors pre and post sequencing and explore genetic testing impact on post imaging behavior | 2009 – most recent data available |
| NLCHI | Meditech – Laboratory Data | PSA testing  FIT testing | Number pre and number post genetic testing  To describe lab utilization behaviors pre and post sequencing and explore genetic testing impact on post lab utilization | 2009 - most recent data available |
|  |  | Year and month of all imaging procedures and lab testing noted in the preceding 2 rows | To explore genetic testing impact on post imaging and lab testing behaviors | 2001-most recent data available |
| NLCHI | MCP Fee-for-Service & PDAD | BRCA Mutation | The presence of this condition will help in understanding how a diagnosed genetic mutation is related (or not) to the need for genetic testing and its relation to health utilization outcomes | 2001 – most recent data available |
| NLCHI | MCP Fee-for-Service & PDAD | Lynch syndrome Mutation | The presence of this condition will help in understanding how a diagnosed genetic mutation is related (or not) to the need for genetic testing and its relation to health utilization outcomes | 2001-most recent data available |
| NLCHI | Meditech | Number and type of specialist appointments | Number pre and post genetic testing  To describe specialist appointments pre and post testing and explore genetic testing impact on utilization of specialists | 2001-most recent data available |
| NLCHI | Meditech | Year and month of all specialist appointments | To describe specialist appointments pre and post testing and explore genetic testing impact on utilization of specialists | 2001-most recent data available |
| Eastern Health | Cancer care registry | Any cancer diagnosis captured in the registry (recognizing not all are, such as blood cancers) | Cancer diagnoses will be explored as potential predictors or confounds of undergoing genetic testing.  Identification of cancer diagnoses posttest results will be explored as a potential impact of having had genetic testing.  For any cancer diagnosis captured in the registry, we need the primary site, stage, and date of diagnosis. | 2001-most recent data available |
| Eastern Health | Cancer care registry | Primary tumor site | To determine any relation between genetic testing and better identification of cancer diagnosis | 2001-most recent data available |
| Eastern Health | Cancer care registry | Stage | To determine any relation between genetic testing and better identification of cancer diagnosis (e.g., lower stage) | 2001-most recent data available |
| Eastern Health | Cancer care registry | Date of diagnosis | To determine any relation between genetic testing and better identification of cancer diagnosis | 2001-most recent data available |
| Eastern Health | Cancer care registry | Smoking history | Smoking history will be needed as it may be possible that the data from NLCHI is not accurate and this data from Eastern Health help us to fill the gaps | 2001-most recent data available |
