# Supplementary material for: Cancer prevention in cancer predisposition syndromes: A protocol for testing the feasibility of building a hereditary cancer research registry and nurse navigator follow up model
Source: PLoS One. 2022 Dec 22;17(12):e0279317. doi: 10.1371/journal.pone.0279317 (PMC9778977; doi:10.1371/journal.pone.0279317)
Supplement: S2 File — (DOCX) [file pone.0279317.s002.docx]

**Interview Guide for hereditary cancer mutation carriers**

Open-ended questions are designed to elicit commentary on experiences with **cancer risk in the family** (first awareness of hereditary link, perceived personal risk,) and **experiences of risk management** (decision-making, understanding implications). A key part of the interview is devoted to patient opinions and preferences regarding the **patient navigation model** for ongoing risk management to help inform the future grant application that will test a pilot navigation project.

**1. Please tell me about your experience with discovering the family’s history of inherited cancer.**

| **Questions:** | **Prompts:** |
| --- | --- |
| Can you tell me how you came to discover your family’s risk for hereditary cancer? | How did you react when you were told you had a cancer predisposition syndrome (name BRCA or Lynch, etc. as appropriate)?  Can you tell me how you felt when you began to realize the implications of this risk for yourself? |
| Can you tell me about what happened next when you realized you were at risk for hereditary cancer? | Were you referred to provincial medical genetics for counseling and testing? How long were you waiting to speak to a counselor? How long did it take to get your test results? Tell me how you felt while waiting to get your test result? |

**2. Can we talk about what having hereditary cancer means for your health (and healthcare).**

| **Questions:** | **Prompts:** |
| --- | --- |
| What recommendations were given to you after being diagnosed with hereditary cancer? | Was any kind of screening recommended? Were appointments made with specialists? Who talked with you about these? Was genetic testing of other relatives recommended? |

**3. I would like to talk about how you manage your cancer risk.**

| **Questions:** | **Prompts:** |
| --- | --- |
| What kinds of things have you been doing to manage your cancer risk? | Do you do any regular screening like colonoscopy, endoscopy, urological screening, pelvic exam, endometrial biopsy, transvaginal ultrasound, preventative surgery? [modify as per what the risk management guidelines are for men/women; specific cancer syndrome].  What is challenging about inherited cancer risk management for you? Are you dealing with any other health conditions that might affect how you manage your inherited cancer risk? |
| Did you feel you had enough information to make an informed decision about how you have decided to manage your cancer risk? | Was there any information you wished you would have had earlier? Or information you felt you didn’t get at all? How did you get your information, from whom? |

**4. Healthcare needs**

| **Questions:** | **Prompts:** |
| --- | --- |
| Have there been any barriers to receiving the screening that has been recommended to you?  Have there been facilitators – things that helped you do the things recommended to you to manage your risks? | Is there anything you feel you need in relation to managing your risks due to hereditary cancer, but have difficulty accessing?  Have you ever received conflicting information about recommended screening? |

**5. Patient navigation models**

Patient navigation programs are patient-centered programs that aim to reduce the lack of coordination and barriers individuals face in navigating the healthcare system. We are wondering what you think about having a nurse act as a navigator for patients in an ongoing way. This would include regular contact with patients, reminding them about screening or other testing appointments, assisting with coordination of screening, helping to provide information of any kind (e.g., social support, community supports, help with genetic testing of children/other family members, etc.) as patients ask for it. We have patient navigators in our cancer care program in NL, but not specifically for people who have a mutation for a cancer gene. It is only when a cancer is diagnosed that patients can work with a navigator. Our hope is to create a model like this for individuals at high risk for cancer because of a hereditary cancer syndrome, but we would like to have patient input to help inform how that might look.

| **Questions:** | **Prompts:** |
| --- | --- |
| Do you think you would like to have access to a patient navigator as we’ve described? | What do you think the advantages of this would be? Would you be okay with a nurse serving in that role? Any other healthcare provider come to mind?  Do any immediate concerns come to mind about a nurse navigator?  At what point in your health care do you think access to a patient navigator would be important?  Would there be an advantage in accessing a navigator before receiving genetic testing results? |
| What kinds of elements do you think a nurse navigator model should have? | Screening reminders? Coordination of screening? Help with emotional/psychosocial support? Regular contact even if there is no upcoming screening? Available at certain times to take phone calls? Assistance with communicating risk within the family? Advice on genetic testing for children or other relatives such as brothers or sisters?  Please tell us anything that comes to mind. |
| Would you be comfortable with a nurse navigator having access to your medical records and communicating with your doctors? | What is concerning about this? If anything?  Would you expect to give informed consent to take part in this kind of model before anything started? How/should your family doctor be involved? |
| Do you have any suggestions for us about how this model should look? | Who should be responsible for it? How should high risk individuals be informed about it? Should there be online elements (e.g., a patient portal where screening tests and reminders are automatically set up?) Any thoughts that come to mind are welcome. |

Please feel free to share any other thoughts you have on managing inherited cancer risk.

Is there something you would like to share or raise that we have not discussed in our interview?

We are nearing the end of our interview. I’d like to gather some demographic information to help describe our study sample. These items are used only for descriptive purposes and are reported as percentages. For example, Xx% of our interview participants are female, XX% are from the Western health authority, XX% are affected by a Lynch syndrome mutation. As before, you can feel free to answer as many or as few as you wish.

**DEMOGRAPHIC ITEMS QUALITATIVE INTERVIEWS**

**1. What is your age?**
 Age______ ☐ Prefer not answer

**2. How do you currently identify in terms of your gender?**
 ☐ Man ☐ Genderqueer/Gender non-conforming
 ☐ Woman ☐ Trans man

☐ Different Identity ☐ Trans woman

☐ Prefer not answer
**3. Do you have a family history of genetic disease?**
 ☐ Yes (please specify) __________

☐ No
 ☐ Prefer not to answer

**4. Which hereditary cancer syndrome do you have?**

☐ Hereditary Breast and Ovarian Cancer Syndrome (*BRCA1*, *BRCA2*)

☐ Lynch Syndrome (*EPCAM*, *MSH2*, *MSH6*, *MLH1*, *PMS2*)

☐ Other __________________

☐ Prefer not to answer

**5. Have you ever been diagnosed with cancer before?**

☐ Yes (please specify) _________
 ☐ No
 ☐ Prefer not to answer

**6. If you answered yes to question 5, at what age were you diagnosed with cancer?**

**7. Do you participate in cancer screening?**

☐ Yes (please specify) __all recommended _for LS carriers______

☐ No
 ☐ Prefer not to answer

**8. What is your marital status? (Please check only one)**
 ☐ Single (never married, no live-in partner)
 ☐ Married
 ☐ Common law/live-in partner
 ☐ Separated/Divorced
 ☐ Widowed
 ☐ Other (please specify): ______________________________________

**9. Do you have any children?**
 ☐ Yes

☐ No

☐ Prefer not to answer

**10. What is the highest level of education that you have completed?**

**(Please check only one)**
 ☐ Less than high school (no certificate, diploma or degree)
 ☐ High school certificate or equivalent
 ☐ Community college, technical college, or CEGEP
 ☐ University – undergraduate degree
 ☐ University – graduate or professional degree
 ☐ Other education or training (please specify): _____________________________

**11. What is your current primary work or employment status?**

**(Please check only one)** ☐ Working full-time ☐ Unemployed, seeking work
 ☐ Working part-time ☐ Retired
 ☐ Volunteer for no pay ☐ Prefer not to answer
 ☐ On temporary disability ☐ On permanent/long-term disability

☐ Work in home/housework

☐ Something else, please specify: ____________________________

**12. What health authority do you currently reside in?**

☐ Eastern

Central

Western

Labrador Grenfell
